# Intranasal Delivery of Collagen-Loaded Neprilysin Clears Beta-Amyloid Plaques in a Transgenic Alzheimer Mouse Model

**DOI:** 10.3389/fnagi.2021.649646

**Published:** 2021-04-22

**Authors:** Christian Humpel

**Affiliations:** Laboratory of Psychiatry and Experimental Alzheimer’s Research, Department of Psychiatry and Psychotherapy, Medical University of Innsbruck, Innsbruck, Austria

**Keywords:** Alzheimer, beta-amyloid plaques, neprilysin, organotypic brain slices, platelets, collagen hydrogel, intranasal

## Abstract

Alzheimer’s disease (AD) is pathologically characterized by extracellular beta-amyloid (Aβ) plaques and intraneuronal tau tangles in the brain. A therapeutic strategy aims to prevent or clear these Aβ plaques and the Aβ-degrading enzyme neprilysin is a potent drug to degrade plaques. The major challenge is to deliver bioactive neprilysin into the brain via the blood-brain barrier. The aim of the present study is to explore if intranasal delivery of neprilysin can eliminate plaques in a transgenic AD mouse model (APP_SweDI). We will test if collagen or platelets are useful vehicles to deliver neprilysin into the brain. Using organotypic brain slices from adult transgenic APP_SweDI mice, we show that neprilysin alone or loaded in collagen hydrogels or in platelets cleared cortical plaques. Intransasal delivery of neprilysin alone increased small Aβ depositions in the middle and caudal cortex in transgenic mice. Platelets loaded with neprilysin cleared plaques in the frontal cortex after intranasal application. Intranasal delivery of collagen-loaded neprilysin was very potent to clear plaques especially in the middle and caudal parts of the cortex. Our data support that the Aβ degrading enzyme neprilysin delivered to the mouse brain can clear Aβ plaques and intranasal delivery (especially with collagen as a vehicle) is a fast and easy application. However, it must be considered that intranasal neprilysin may also activate more plaque production in the transgenic mouse brain as a side effect.

## Introduction

The life expectancy of humans has markedly increased over the last 100 years. As age is the main risk factor for Alzheimer’s disease (AD), the number of patients suffering from AD and mixed forms of dementia will dramatically increase over the next 50 years. It is expected that there will be about 80 million AD patients worldwide in 2050. AD is characterized by severe β-amyloid (Aβ) deposition in brain (extracellular plaques), tau pathology (hyperphosphorylated tau causes neurofibrillary tangles; NFT), cell death of cholinergic neurons (loss of the neurotransmitter acetylcholine), astroglial and microglial activation, inflammation and cerebrovascular damage.

Amyloid-precursor protein (APP) is a large transmembrane protein and secretases process different Aβ peptides. Especially the 42 amino acid Aβ peptide is toxic and can aggregate and form the typical plaques in the AD brain. The plaques consist of a central core of highly aggregated Aβ peptides and a halo around the plaques (Seeman and Seeman, 2011; Daschil and Humpel, 2016), which contains degenerating nerve fibers and several infiltrating cells, like reactive astrocytes or microglia. In severe AD, the whole brain is filled with several Aβ plaques. Therapeutic options aim to prevent the deposition of plaques using beta-sheet breaker, secretase inhibitors, or Aβ-degrading enzymes to degrade and clear the plaques (Glabe, 2000; Wang et al., 2006). It is known that infusion of plaque-degrading enzymes may be an attractive strategy to destruct plaques. Indeed, several Aβ-degrading proteases have been identified, including neprilysin (NEP), insulin-degrading enzyme, endothelin-converting enzyme, angiotensin-converting enzyme, plasminogen activators, or different matrix metalloproteinases.

Neprilysin is a 90–110 kDa plasma membrane glycoprotein of the neutral (M13) zinc metalloendo-peptidase family expressed by neurons and cerebrovascular smooth muscle cells and is the most potent Aβ-degrading enzyme (Iwata et al., 2001). NEP is identical with the neutrophil cluster differentiation antigen CD10, and is also known as the common acute lymphoblastic leukemia antigen (CALLA). Several members have been identified, composed of a short N-terminal cytoplasmic region, a membrane spanning section and a large C-terminal extracellular, catalytic domain which contains the typical HExxH zinc binding motif (Carson and Turner, 2002). NEP is an ectoenzyme preferentially hydrolyzing extracellular oligopeptides (< 5kDa) on the amino side of hydrophobic residues. Indeed, several cleavage sites of NEP within the Aβ sequence have been identified in human and mouse (Carson and Turner, 2002; Grimm et al., 2013): Glu_3_/Phe_4_, Gly_9_/Tyr_10_ (human only), Phe_19_/Phe_20_, Ala_30_/Ile_31_, and Gly_33_/Leu_34_. NEP has been shown to cleave at these sites in a phosphoramidon-sensitive manner with a K_m_ of 2.8 μM for Aβ_42_ (Carson and Turner, 2002). Intracerebral infusion of a recombinant soluble neprilysin from insect cells into AD mice improved memory and reduced Aβ accumulation in the brain (Park et al., 2013). Further an intravenous infusion of a NEP resulted in dose-dependent clearance of Aβ (Walker et al., 2013). Miners et al. (2011) showed that NEP counteracted Aβ-induced toxicity. In a previous study we showed that recombinant NEP degraded Aβ plaques in organotypic brain slices taken from adult transgenic AD mice (Humpel, 2015a,b).

However, Aβ-degrading enzymes are large molecules, are unstable in blood and cannot pass the blood-brain barrier (BBB) and do not find their targets easily. Thus, different strategies need to be tested, to deliver stable neprilysin directly into the brain, especially *in vivo* in AD mouse models. One approach uses intracranial injection directly into the brain (Park et al., 2013; Walker et al., 2013) with many severe surgical side effects. Thus in order to test a safe and non-invasive delivery blood cells as vehicles are of interest. In fact, we have shown that platelets (thrombocytes) (Humpel, 2017) can be effectively loaded with protective proteins (Kniewallner et al., 2014) and migrate into the brain after intravenous infusion (Kniewallner et al., 2016). However, we found that the majority of migrated cells was captured in the periphery, like spleen or liver, and other alternative approaches must be tested, to deliver substances directly to the brain, such as e.g., intranasal delivery. Another approach uses biomaterials to encapsulate protective substances in order to provide a stable and slow release. In fact, we have shown that collagen is a very potent natural biomaterial and growth factors, such as NGF (Foidl et al., 2018), or GDNF (Ucar et al., 2021) or FGF-2 (Ucar et al., 2020) can be loaded and applied on brain slices.

Already in 1933 the first report was published on intranasal instillation (Faber and Gebhardt, 1933), which entered therapeutic interventions several decades later (see review Podolska et al., 2012). Nowadays mainly intranasal insulin treatment is the best published compound regarding AD, as it improves memory and learning in AD models (Reger et al., 2008; Farzampour et al., 2016; Mao et al., 2016; Guo et al., 2017), it lowers Aβ in diabetes models (Subramanian and John, 2012), it modulates verbal memory (Reger et al., 2008) or it prevents tau phosphorylation in 3xTg mice (Chen et al., 2014) and type II diabetes (Yang et al., 2013). Interestingly, also other intranasal substances had effects in AD models, as they counteracted Aβ toxicity (Maurice et al., 2013; Kaur and Prakash, 2019), decreased Aβ and inflammation (Lin et al., 2016; Drews et al., 2019), affected APP processing (Guo et al., 2017), prevented memory deficits (Fine et al., 2012; Lou et al., 2016; Rodríguez Cruz et al., 2017), reduced aggregation of Aβ and tau in AD mice (Matsuoka et al., 2007) or decreased tau phosphorylation in alpha-synuclein mice (Magen et al., 2014). It is noteworthy that intranasal infusion of the most potent cholinotrophic substance nerve growth factor (NGF) prevented memory deficits in AD mice (Capsoni et al., 2012), or ameliorated Aβ deposition (Tian et al., 2012) or attenuated tau phosphorylation (Lv et al., 2014). Taken together, there is clear evidence that intranasal application is non-invasive, cheap and easy to apply and applied substances can reach the brain and counteract some AD pathologies.

The aim of the present study was (1) to strengthen our previous findings (Humpel, 2015b) that neprilysin can clear plaques in adult brain slices, and test if a collagen hydrogel can provide a more stable application form, (2) to explore if platelets loaded with neprilysin can be an alternative strategy to clear plaques in brain slices, and (3) finally to translate these findings into *in vivo* mouse models and test if intranasal applications can clear plaques in transgenic AD mice.

## Materials and Methods

### APP_SDI Mice and Controls

In this study we used wildtype (C57BL/6N) and transgenic APP_SDI (expressing amyloid precursor protein (APP) harboring the Swedish K670N/M671L, Dutch E693Q, and Iowa D694N mutations; C57BL/6-Tg (Thy1-APPSwDutIowa)BWevn/Mmjax) mice, which were housed at the Medical University of Innsbruck animal facility providing open access to food and water under 12 h/12 h light-dark cycles. The transgenic mice produce Aβ plaques at an age >6 months (Davis et al., 2004) and we have long experience with this transgenic AD mouse model (Daschil and Humpel, 2016). All experiments conformed to Austrian guidelines on the ethical use of animals and all efforts were made to minimize the number of animals used and their suffering and were approved by the Austrian Ministry of Science (Nr 2020-0.432.218).

### Organotypic Brain Slices

**FIGURE 1:**
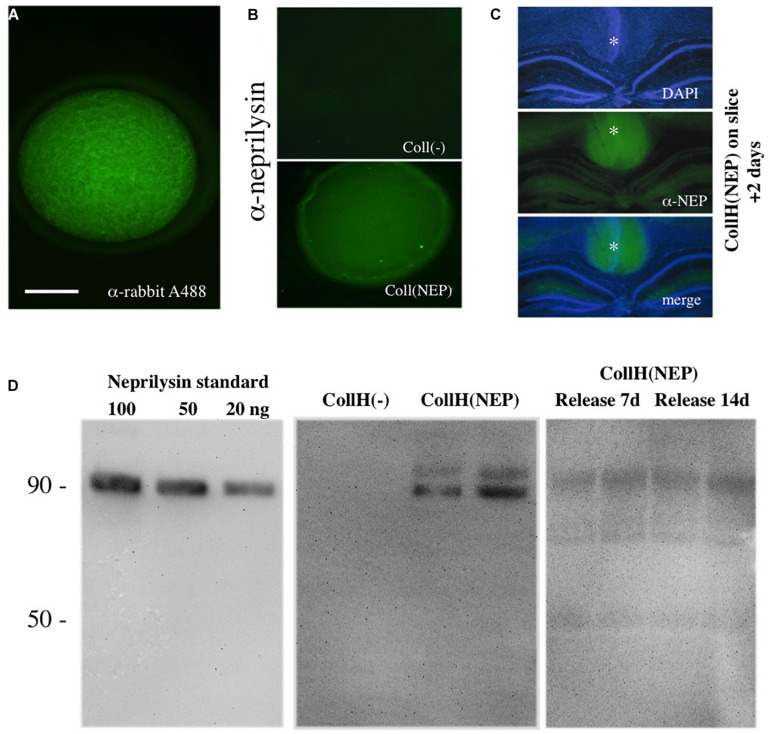
Characterization of neprilysin-loaded hydrogels. A collagen hydrogel can be visualized under the fluorescence microscope after loading with a rabbit-Alexa 488 antibody **(A)**. Immunostainings with a neprilysin antibody (Alexa 488, green) shows successful loading of recombinant neprilysin into collagen hydrogels, compared to a negative hydrogel **(B)**. When neprilysin-loaded collagen hydrogels were loaded onto slices (as indicated by a *), incubated for 2 days and then fixed and stained for neprilysin, the hydrogels (middle, Alexa-488, green fluorescent) were clearly visible above the DAPI-counterstained (blue florescent, upper panel) hippocampal formation **(C)**. Western Blot Analysis shows detection of neprilysin with a detection limit of at least 20 ng/lane with a size of approx. 90 kDa **(D)**. Collagen hydrogels loaded with [CollH(NEP)] or without [CollH(−)] neprilysin show successful loading in hydrogels **(D)**. Release experiments show that very low amounts of neprilysin are released into the medium after 7 or 14 days **(D)**. Scale bar in **(A)** = 600 μm **(A)**, 800 μm **(B)**, and 1400 μm **(C)**.

For brain slice cultures 9 month old adult mice were used and organotypic brain slices were performed as described in detail (Humpel, 2015a,b, 2018). The animals were rapidly sacrificed, the brains dissected and coronally cut. The brains were glued (Glue Loctite) onto the chuck of a water cooled vibratome Leica VT1000A (UV sterilized and under a Laminar Flow), and triggered close to a commercial shave racer. Under aseptic conditions, 110 μm thick coronal slices at the hippocampal level were cut and collected in sterile medium. The organotypic slices were carefully placed onto a sterile 0.4 μm pore membrane (Millipore HTTP02500), which was then placed into a 0.4 μm membrane insert (Millipore PICM03050) within a 6-well plate (see Figure 1A). Vibrosections were cultured in 6-well plates (Greiner) at 37°C and 5% CO_2_ with 1.2 ml/well of the following culture medium: 50% MEM/HEPES (Gibco), 25% heat inactivated horse serum (Gibco/Lifetech, Austria), 25% Hanks’ solution (Gibco), 2 mM NaHCO3 (Merck, Austria), 6.5 mg/ml glucose (Merck, Germany), 2 mM glutamine (Merck, Germany), pH 7.2. In order to study clearance of plaques, neprilysin alone, or neprilysin loaded into collagen hydrogels, or neprilysin loaded into platelets were applied to the brain slices and incubated for 2 weeks, then postfixed 3 h with 4% paraformaldehyde and then stained by immunohistochemistry.

### Neprilysin

Recombinant human neprilysin/CD10 was purchased from R&D (Nr. 1182-ZNC-010), dissolved in 100 μl sterile a.d. (100 μg/ml), aliquoted and stored frozen at minus 80°C; at the day of use, aliquots were thawed and directly used for the experiment.

### Collagen Hydogels

In order to guarantee a slow, stable and local application of substances, neprilysin was loaded in collagen-loaded hydrogels. We have extensive experience in applying substances onto brain slices using non-toxic collagen hydrogels, which degrade over time and release the factor of interest (Foidl et al., 2018; Ucar et al., 2020, 2021). Briefly, 0.4 mg bovine collagen type I (Collagen Solutions^®^, United Kingdom) was linked with 0.8 mg 4S-StarPEG in phosphate buffered saline (PBS) at pH 7.4, obtaining a final hydrogel of 2 mg/ml collagen and 1:2 molar ratio of coll:4S-StarPEG. Drops of 2 μl were pipetted onto Teflon tapes and gelled for 1 h at 37°C.

When loading NEP, 10 μl of a peptide stock (1 μg) was added to the collagen hydrogels before crosslinking. Hydrogels (2 μl drops) have a size of approx. 1–2 mm; these hydrogels can be easily handled (see Foidl et al., 2018 for details) and placed onto brain slices or into the nose. In order to visualize the hydrogels 10 μl of fluorescent Alexa-488 anti-rabbit antibodies were loaded before crosslinking. In order to visualize neprilysin in hydrogels, hydrogels were loaded with recombinant neprilysin, immediated fixed with 4% paraformaldehyde and then stained by immunohistochemistry. For the release experiments neprilysin-loaded hydrogels (300 ng neprilysin in total) were placed on small parafilms and then incubated in slice medium for 2, 7 and 14 days and the supernatants analyzed by Western Blot.

### Isolation of Platelets and Loading With Neprilysin

Wildtype C57BL6 mice (6 months old) were anesthetized with ketamine (100 mg/kg) and Xylazine (10 mg/kg) and blood was directly drawn from the heart by using a 21 gauge butterfly blood collection system (BD Valu- Set, BD). The blood was collected in ethylenediaminetetraacetate (EDTA) tubes (S-monovettes, Sarsted) and gently mixed. Platelets were isolated as described in detail (Kniewallner et al., 2014) and characterized using FACS analysis for CD61, a specific antigen that is expressed on the surface of resting platelets. Immediately after blood collection, anticoagulated blood was centrifuged at 100×*g* for 10 min to obtain platelet rich plasma (PRP). All centrifugation steps were performed at room temperature. PGI_2_ (Prostaglandin, 500 nM, Sigma) was added to prevent platelet activation during processing. Platelets were separated from PRP by centrifugation at 400×*g* for 10 min and were either dissolved in 10 μl PBS or 10 μl neprilysin stock solution (1 μg neprilysin/10 μl). To load neprilysin into platelets, platelets were then incubated with neprilysin in an ultrasound path (Bandelin Sonorex RK514, power 215; 860W, 35 kHz frequency) for 3 h on ice as reported by us (Kniewallner et al., 2014). Then 200 μl PBS was added to the platelets, centrifuged again at 400×*g* 10 min and the pellet dissolved in 80 μl PBS and used for application on organotypic brain slices or intranasal application. The efficiency to load substances with ultrasound has been shown to be ca. 30% (Kniewallner et al., 2014). In each nose we applied 10 μl solution with 5 million platelets + 40 ng neprilysin.

### Intranasal Delivery

**FIGURE 2:**
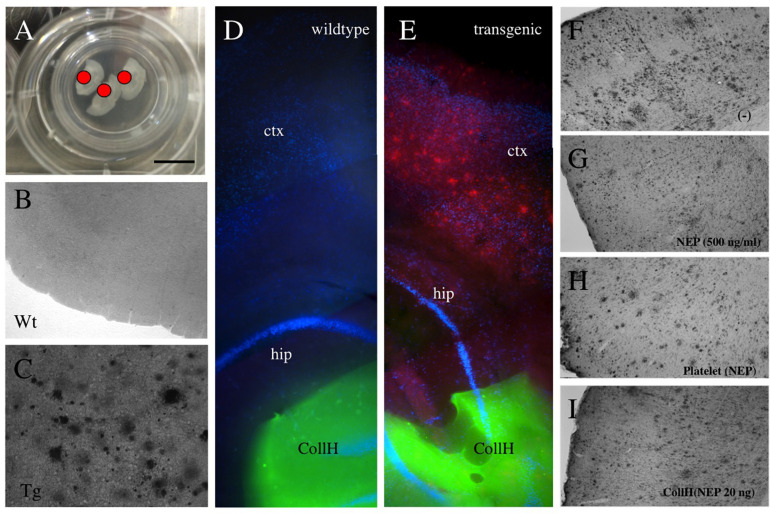
Application of collagen- hydrogels on organotypic brain slices. Organotypic brain slices (110 μm thick, 3 brains per well) were prepared and cultured on semipermeable membranes **(A)** and hydrogels were applied directly onto the brain slice [red dot in **(A)** and green fluorescent in **(D,E)**]. Collagen hydrogels were applied onto 9 month old wildtype mice brain slices **(B,D)** or slices from transgenic APP_SweDI mice with plaques **(C,E)**. No plaques are seen by immunostainings in wildtype mice **(B,D)**, while in transgenic mice many small and large **(C,E,F)** plaques are visible. Neprilysin [500 ng/ml in medium; **(G)**], loaded into platelets **(H)** or loaded into hydrogels **(I)** reduced the plaque number, compared to a negative control **(F)**. Scale bar in A = 7.5 mm **(A)**, 200 μm **(B,C)**, 500 μm **(D,E)**, 400 μm **(F–I)**. ctx, cortex; hip, hippocampus; Wt, wildtype; Tg, transgenic; CollH, collagen hydrogels; NEP, neprilysin.

Mice were narcotized with Ketamine (100 mg/kg) and Xylazine (10 mg/kg). Ten μl substances were applied 5 mm deep bilaterally into the nose via the nostril (naris) using magnification glasses and a bright light using a 10 μl pipette (white tips) (see Figure 2A). The substances were slowly infused in PBS-EDTA-heparin. Alternatively, a collagen hydrogel (with 30 ng neprilysin per hydrogel) was applied directly into the nose bilateral. Animals were allowed to recover. The application was repeated on days 2 and 3. After 6 days of recovery (9 days in total) mice were transcardially perfused with 4% paraformaldehyde and brains collected and postfixed (3 h), frozen in a CO_2_ stream, cryosectioned (40 μm) and stained by immunohistochemistry. As a control and to optimize the intranasal delivery (a) blue fluorescent Aβ (human Aβ_42_, AMCA-LC labeled, ANASpec 60475-01; Aβs/Em = 354/442 nm) was infused and analyzed after 24 h or (b) a cresyl violet saturated solution was infused and analyzed after 3 h. Brains were taken, frozen in a CO_2_ stream, cryosectioned and visualized under the fluorescence microscope (Aβ) or light microscope (cresyl violet) or counterstained with blue DAPI (fluorescence microscope, Leica DM IRB).

### Western Blots

Collagen hydrogels, neprilysin standards (20-50-100 ng/lane) or medium with released neprilysin (20 μl per lane) were denatured (10 min 70°C with reducing agent) and loaded onto 10% Bis-Tris polyacrylamide gel (Invitrogen) and electrophoresis was performed for 35 min at 200 V. Samples were electrotransferred onto PVDF membranes for 20 min at 25 V in a semi-dry transfer cell (Thermo Fisher Scientific). Blotting was done by using WesternBreeze Chemiluminescent immunodetection system (Invitrogen). Blots were blocked with blocking buffer for 30 min and incubated overnight on a shaker at 4°C with primary antibodies against neprilysin (abcam ab210932; 1:1000). Subsequently, blots were briefly washed and incubated with alkaline phosphatase-conjugated secondary mouse (neprilysin) for 30 min at room temperature. Afterward, the blots were briefly washed again, incubated for 15 min in CDP-Star chemiluminescent substrate solution (Roche) and visualized with a cooled CCD camera (SearchLight, Thermo Fisher Scientific).

### Immunohistochemistry

Immunohistochemistry was performed as previously described under free-floating conditions (Humpel, 2015b). The slices were washed with PBS and incubated in PBS/0.1% Triton (T-PBS) for 30 min at 20°C while shaking. To quench endogenous peroxidase, sections were treated with PBS/1%H_2_O_2_/5% methanol. After incubation, the sections were then blocked in T-PBS/20% horse serum (GIBCO Invitrogen)/0.2% BSA (SERVA) for 30 min at 20°C shaking. Following blocking with mouse IgG blocking reagent (Vector MKB-2213), brain sections were incubated with primary antibody against beta-amyloid (1-16; 6E10; Covance SIG-39300) or neprilysin (abcam 210932; 1:200) in T-PBS/0.2% BSA overnight at 20°C. The sections were then washed and incubated with a biotinylated secondary mouse antibody (1:200, Vector Laboratories) in T-PBS/0.2% BSA for 1 h at 20°C shaking. Following secondary antibody incubation, sections were rinsed with PBS and incubated in avidin-biotin complex solution (Elite ABC kit, Vector Laboratories) for 1 h at 20°C shaking. Finally, the sections were washed with 50 mM Tris-buffered saline (TBS) and then incubated in 0.5 mg/ml 3,3’-diaminobenzidine (DAB, Sigma)/TBS/0.003% H_2_O_2_ at 20°C in the dark until a signal was detected. Once DAB staining was visible, the reaction was stopped by adding TBS to the sections. The brain sections were rinsed with TBS, inserted into a 6-well plate (slice down to well surface) directly onto a drop of Vectashield (Vector), cover-slipped and then evaluated under an inverse microscope (Leica DM IRB). For neprilysin the stainings were visualized using anti-mouse Alexa-488 antibody and brain slices counterstained with nuclear blue fluorescent DAPI. In addition, plaques were also stained by thioflavin S (2 μg/ml, overnight) and counterstained with nuclear DAPI.

### Evaluation of Cortical and Hippocampal Plaques

Brain sections at the cortical level were analyzed by a blinded evaluator as reported previously (Humpel, 2015b). Briefly sections were photographed with the Leica inverse microscope at a 10× magnification under a red filter. The exposure time was always 23 ms with the bright light set at the lowest level. The software Openlab was used at a Mac computer connected to the microscope. Pictures were saved as JPG files and the analysis was performed using Image J. The pictures were transformed to a 8-bit grayscale image. The calibration was set at 0.470 (distance in pixels), 1.00 (known distance), 1.0 (pixel aspect ratio) and μm (unit in length) and global was activated. The picture was transformed into a binary image and the threshold was adapted to 30–40. The number of particles was counted setting the size to 100–8000 pixels. For some sections small (100–400 pixel), medium (400–1000 pixel) and large (1000–8000 pixel) depositions were counted. The number of plaques was counted in a defined circle in 2.5 mm^2^ area.

### Statistical Analysis

Statistical analysis was performed by a One Way ANOVA with a subsequent Fisher LSD *post hoc* test where *p* < 0.05 represents significance.

## Results

### Characterization of Neprilysin-Loaded Hydrogels

Collagen hydrogels were visualized by loading fluorescent Alexa-488 antibodies and were found as ca. 2 mm round pieces (Figure 1A). In order to visualize hydrogels loaded with neprilysin, immunostainings were performed and showed recombinant neprilysin in hydrogels (Figure 1B), compared to a negative control (Figure 1B). When neprilysin-loaded collagen hydrogels were loaded onto slices, incubated for 2 days and then fixed and stained for neprilysin, the hydrogels were clearly visible above the hippocampal formation (Figure 1C). Western Blots were used to characterize recombinant neprilysin as a 90 kDa protein, and the detection limit was around 5–20 ng/lane (Figure 1D). In order to characterize loading of neprilysin into hydrogels, Western Blot verified the protein, with a size of 90 kDa, and a slightly larger band was also visible (Figure 1D). Release experiments showed that the release into medium was very low after 7 and 14 days (Figure 1D).

### Neprilysin Clears Plaques in Adult Brain Slices

Adult organotypic brain slices were cultured for 2 weeks on semipermeable membrane inserts (Figure 2A). The hydrogels were placed directly on the slices of wildtype (Figure 2D) or transgenic (Figure 2E) mice. Approximately 280 plaques were counted in slices taken from transgenic mice (Figures 2C,E and Table 1), while no plaques were seen in wildtype mice (Figures 2B,D and Table 1). Neprilysin significantly decreased the number of plaques when added at a concentration of 20 ng/ml or 500 ng/ml to the medium (Table 1 and Figures 2F,G). Neprilysin loaded to collagen hydrogels decreased the number of cortical plaques when added at a concentration of 20 ng/hydrogel (Table 1 and Figure 2I), but not at lower concentrations of 2 and 0.2 ng/hydrogel (Table 1).

**TABLE 1:** Quantification of beta-amyloid plaques in brain slices treated with neprilysin alone or loaded in collagen- hydrogels.

			***p*-values**	

	**Plaques/field**	**vs WT(−)**	**vs TG(−)**	**vs TG(CollH)**
WT (−)	0 (4)	–		
WT + 20 ng/ml NEP medium	0 (4)			
WT + CollH (−)	0 (5)			
TG (−)	281 ± 38 (9)	***	–	
TG + 20 ng/ml NEP medium	97 ± 8 (8)		**	
TG + 500 ng/ml NEP medium	35 ± 4 (8)		***	
TG + CollH (−)	192 ± 30 (15)		ns	–
TG + CollH NEP (0.2 ng)	221 ± 30 (6)			ns
TG + CollH NEP (2 ng)	251 ± 35 (6)			ns
TG + CollH NEP (20 ng)	56 ± 9 (24)			***

Adult brain slices from 9 month old transgenic (TG) transgenic Alzheimer mice or C57BL6 wildtype mice (WT) were incubated with or without neprilysin (NEP) in medium (0-20-500 ng/ml) or loaded in collagen hydrogels (0-0.2-2-20 ng/2 μl hydrogel) and applied onto the brain slice. After 2 weeks slices were fixed and immunohistochemically stained for beta-amyloid. The number of plaques was counted using computer assisted imaging on 3 fields of the cortex and averaged. Values are given as mean ± SEM (n = number of mice); statistical analysis was performed by one Way ANOVA with a subsequent Fisher LSD *post hoc* test (**p < 0.01; ***p < 0.001; ns, not significant).

### Platelets Loaded With Neprilysin Clear Plaques in Adult Brain Slices

In the next step we wanted to show if neprilysin loaded to platelets can also clear the plaques in the slices taken from adult transgenic mice. In this experiment we quantified small, medium and large plaques, because we expected less effects. In control slices 75 small, 26 middle sized and 12 large plaques were found per field (Table 2). This number did not change when platelets alone or platelets loaded with a negative empty control were added (Table 2). When platelets were loaded with neprilysin by ultrasound, the number of small and middle-sized but not large plaques was significantly reduced (Table 2 and Figure 2H). Sonication of these platelets slightly reduced the effects (Table 2).

**TABLE 2:** Quantification of beta-amyloid plaques in brain slices treated with neprilysin loaded platelets.

**(*n* = 6)**	**Small**	**Middle**	**Large**
PBS control	7513	263	122
Platelets	7814*ns*	213*ns*	113*ns*
Platelets (-) loaded	8214*ns*	233*ns*	92*ns*
Platelets (NEP) loaded	317*	152**	71*ns*
Platelets (NEP) loaded-SONIC	497*ns*	173*ns*	91*ns*

Platelets were isolated from adult wildtype mice and loaded with or without neprilysin in an ultrasound bath in ice for 3 h resulting in 20 ng neprilysin in 2.5 million platelets per 5 μl (with a loading efficiency of 30%). Adult brain slices (110 μm thick) from 9 month old APP_SweDI mice were cultured and after 1 day 5 μl of platelets (2.5 million platelets + 20 ng neprilysin) were applied on the brain slice. As a control PBS was applied or isolated platelets without loading. As an additional control, NEP-loaded platelets were sonicated. After 2 weeks in culture, brain slices were fixed and stained for beta-amyloid plaques and the number of small (100–400 pixel) - middle (400–1000 pixel) - large (1000–8000 pixel) plaques was counted. Statistical analysis was performed by ANOVA with a subsequent Fisher-LSD *post hoc* test (*p < 0.05; **p < 0.01; ns, not significant).

### Intranasal Delivery of Neprilysin Clears Plaques

**FIGURE 3:**
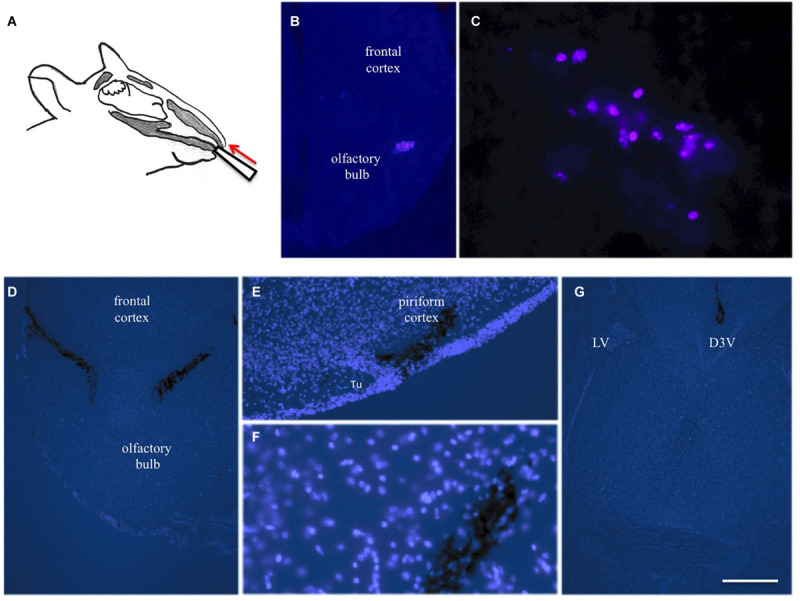
Intranasal infusion into mice. Substances (10 μl) were infused via the left and right nostril in anesthetized mice using a 10 μl pipette **(A)**. Twenty four hours after infusion of blue fluorescent labeled beta-amyloid several blue-labeled cells were seen in the bulbus, indicating phagocytosis of the peptide **(B,C)**. Three hours after infusion of cresyl violet several labeled areas were seen in the bulbus **(D)**, piriform cortex **(E,F)** and hippocampus **(G)**. Brains were counterstained with blue DAPI and pictures are merged with bright-light black labeled cresyl violet. Scale bar in **(G)** = 150 μm **(B,D,G)** and 75 μm **(C,E,F)**. D3V, 3rd ventricle; LV lateral ventricle; Tu, olfactory tubercle.

In the next step we wanted to translate these finding to *in vivo* mice via intransal infusion (Figure 3A). In order to optimize the intranasal delivery, blue fluorescent labeled beta-amyloid was infused and several blue-labeled cells were seen in the bulbus 24 h after infusion (Figures 3B,C). Three hours after infusion of cresyl violet several labeled areas were seen in the bulbus (Figure 3D), piriform cortex (Figures 3E,F), and hippocampus (Figure 3G).

**FIGURE 4:**
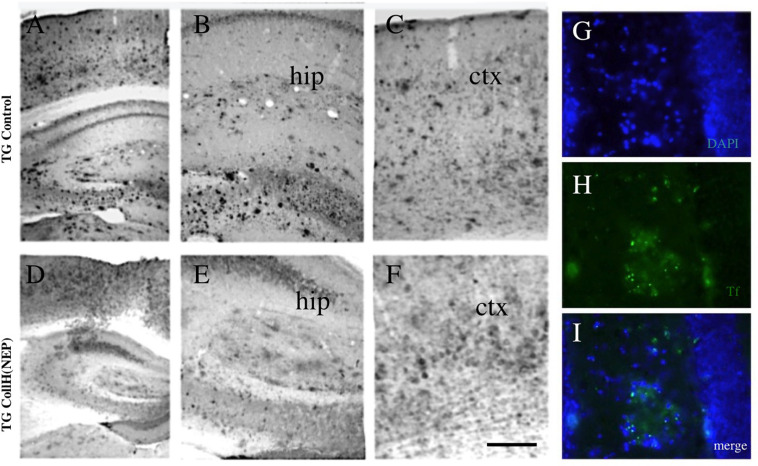
Immunohistochemical staining of brain sections after intranasal delivery of neprilysin. Transgenic mice were intransally infused with a control [TG control; **(A–C)**] or with a collagen hydrogel loaded with neprilysin [TG CollH(NEP); **(D–E)**]. Brains were sectioned and stained for beta-amyloid like immunoreactivity **(A–F)**. Note a strong plaque load in hippocampus [hip, **(B,E)**] and cortex [ctx, **(C,F)**] and a somewhat reduced plaque density in mice treated with neprilysin. As an additional control plaques were stained with thioflavin S [Tf, **(H,I)**] and counterstained with blue fluorescent DAPI **(G,I)**. Scale bar in **F** = 200 μm **(A–F)**; 120 μm **(G–I)**.

In order to see if intransal delivery of neprilysin can clear plaques in the brain, neprilysin was infused directly into the nose of anesthetized mice (repeated on day 1-2-3) and analyzed after 6 days. No small and large plaques were seen in wildtype mice (Table 3). In transgenic mice approx. 100 small plaques and approx. 7 large plaques were found in the frontal, middle and caudal cortex (Table 3 and Figures 4A–C). Repeated infusion of neprilysin alone (100 ng/nose/10 μl, bilateral, on days 1-2-3) slightly reduced the number of small plaques in the frontal cortex but increased small Aβ depositions in the middle and caudal areas (Table 3). When plateletes loaded with neprilysin were infused via the nose to transgenic mice then the number of small and large plaques was cleared but only in the frontal cortex and not in the middle and caudal cortex (Table 3). Application of collagen-loaded neprilysin to the nose of transgenic mice markedly reduced the number of small and large plaques, which was more pronounced in the middle and caudal cortex but not seen in the frontal cortex (Table 3 and Figures 4D–F). The plaques were verified using Thioflavin S stainings and counterstained with nuclear DAPI (Figures 4G–I). In the hippocampus neprilysin-loaded collagen hydrogels (135 ± 10, *n* = 5, *p* = 0.1) did not affect the number of small and large plaques compared to controls (113 ± 11, *n* = 6) as analyzed by computer-assisted analysis (area 2.8 mm^2^; 100–8000 pixels).

**TABLE 3:** Intranasal infusion of neprilysin and quantification of plaques.

**A: SMALL PLQ**	**n**	**Frontal**	**Middle**	**Caudal**
WT	3	0	0	0
TG	6	98 ± 6	90 ± 13	99 ± 13
TG + NEP	5	75 ± 6 (*p* = 0.054)	140 ± 17*	154 ± 20*
TG + NEP(platelet)	6	66 ± 13*	83 ± 16	103 ± 14
TG + NEP(CollH)	5	72 ± 6*	25-1-5***	7.9 ± 1.8***

**B: LARGE PLQ**	***n***	**Frontal**	**Middle**	**Caudal**

WT	3	0	0	0
TG	6	6.8 ± 0.9	4.1 ± 0.6	6.0 ± 0.9
TG + NEP	5	6.3 ± 1.5	6.5 ± 0.8 (*p* = 0.06)	7.5 ± 1.9
TG + NEP(platelet)	6	3.2 ± 0.8*	4.4 ± 1.2	6.7 ± 1.5
TG + NEP(CollH)	5	7.1 ± 0.8	1.5 ± 0.4**	0.4 ± 0.1***

Nine month old wildtype (WT) or transgenic (APP_SweDI, TG) mice were narkotized and 10 μl suspension was applied in the left and right nostril. Animals were allowed to recover and application was repeated on days 2 and 3. Animals received intranasal infusions of neprilysin (10 μl = 100 ng NEP) or NEP-loaded platelets (10 μl = 5 Mill platelets + 40 ng neprilysin with 30% loading efficiency). Alternatively, NEP loaded collagen hydrogels (CollH) were placed in the left and right nose (30 ng NEP/nose) on 3 consecutive days. Animals were taken at day 9, perfused, and brains cryosectioned at 3 Bregma levels (frontal, middle, and caudal) and immunostained for beta-amyloid plaques (PLQ). The number of small/middle (400–1000 pixel) and large (1,000–8,000 pixel) plaques was counted. Statistical analysis was performed by ANOVA with a subsequent Fisher-LSD *post hoc* test (*p < 0.05; **p < 0.01; ***p < 0.001). n gives the number of mice.

## Discussion

In the present study, we show that the Aβ-degrading enzyme neprilysin can clear plaques in an organotypic brain slice model of adult transgenic AD mice. We show that neprilysin can be loaded into collagen hydrogels and that intranasal delivery of the neprilysin-loaded collagen hydrogels eliminates plaques in the caudal cortex *in vivo*.

### Organotypic Brain Slices

Organotypic brain slices are well established 3-dimensional culture models containing the complex interaction of neurons, glia, and vessels and have been extensively used to study the survival of different neurons and thus allow to explore mechanisms in a simple *ex vivo* AD model (Humpel, 2015a,b; Iwata et al., 2001). In our hands, we have optimized and characterized this model and used whole coronal or sagittal vibroslices, containing a more complex interaction of the cells and brain areas (Humpel, 2015a,b; Iwata et al., 2001). Usually most brain slices are derived from postnatal animals, as it is difficult to properly prepare and maintain adult slices. Culturing of adult brain slices is very tricky and has been reported by us (Humpel, 2015b). We have shown that neprilysin can eliminate plaques in such a model of adult transgenic slices. The present study is an extension of the previous one and we further proof again, that pure neprilysin in medium alone (> 20 ng/ml) can clear plaques in such a model. In the present study we wanted to use more advanced methods to optimize the delivery of neprilysin. In order to reach this aim, we (a) used collagen as a stable biomaterial to encapsulate neprilysin and (b) we used platelets as a vehicle to deliver neprilysin to the brain.

### Collagen Hydrogels as a Delivery Model

Collagen and collagen hydrogels have been widely used to repair various tissues and structures including brain (see our review Ucar and Humpel, 2018). Collagen hydrogels loaded with growth factors such as NGF (Foidl et al., 2018), GDNF (Ucar et al., 2021), or FGF-2 (Ucar et al., 2020) have been extensively used and characterized by us on organotypic brain slices. In these studies, we showed that substances can be loaded into collagen hydrogels and be released in a time-dependent pattern, and we showed that growth factor-loaded collagen hydrogels provided neuroprotection to neurons in organotypic brain slices (Foidl et al., 2018). We further showed that these collagen hydrogels were not toxic when applied onto brain slices and exhibited only a minor reactive gliosis (Foidl et al., 2018). In the present study we loaded neprilysin into such collagen hydrogels and placed them onto the brain slices. In fact, we also show that neprilysin released from these collagen hydrogels (20 ng/hydrogel) can clear the plaques. As collagen hydrogels are not toxic and slowly degrade, neprilysin is released and diffuses either on the slices or via the medium and clears the plaques. Thus we provide strong evidence that neprilysin-loaded collagen hydrogels are potent delivery vehicles.

### Platelets Loaded With Neprilysin as a Vehicle

Platelets (thrombocytes) are 3–5 μm small anuclear cells and play a central role in the blood clotting process and repair of vessels but are also affected in AD or cerebral amyloid angiopathy (see my review Humpel, 2017). For platelets characterization we used FACS analysis for CD61, a specific antigen that is expressed on the surface of resting platelets (data not shown). Platelets contain several biogenic substances in their secretory granules, including growth factors. We have previously reported that ultrasound (35 kHz) is suitable to load NGF into platelets and enhance and release bioactive NGF levels sixfold (Kniewallner et al., 2014). In the present study we extend our findings and report for the first time that platelets can also be loaded with neprilysin giving a concentration of 80 ng neprilysin per 10 mill platelets, at a loading efficiency of 30%. Using neprilysin-loaded platelets, we show now that we can also eliminate and clear plaques in our adult transgenic mouse model, being similarly efficient as with neprilysin alone. Thus we provide strong evidence that platelets could be interesting delivery vehicles *in vivo*, as they are very small cells and may pass via the intranasal way.

### Intranasal Delivery Is Potent to Deliver Drugs Into the Brain

In the first step, we tested and optimized the intranasal delivery. Substances (10 μl) were infused via the left and right nostril in anesthetized mice using a 10 μl pipette. Twenty four hours after infusion of blue fluorescent labeled Aβ several blue-labeled cells were seen in the bulbus but not in deeper brain areas. These stainings were visualized intracellular and we conclude that the Aβ peptide was incorporated by phagocyting cells (e.g., microglia). Not many papers have been published demonstrating intranasal delivery of Aβ. The most prominent one is the study of Sipos et al. (2010), who intranasally applied human Aβ(42) in a form of oligomers, protofibrils, and fibrils into rats. Nasally injected Aβ could be detected in the olfactory bulb and frontal cortex but also deeper in hippocampus, pons, and cerebellum 2 h after a single dose (Sipos et al., 2010). As a second control we infused the dye cresyl violet and several labeled areas (counterstained with fluorescent DAPI) were seen in the bulbus, piriform cortex, and hippocampus 3 h after infusion. This clearly shows that dyes are rapidly taken up and transported to the brain and also into deeper brain areas and that indeed intranasal delivery works in our hands.

### Intranasal Delivery of Neprilysin

In the second step, we infused now neprilysin alone using our optimized intranasal delivery system. As a delivery matrix we used PBS-EDTA-heparin, which prevents blood clotting in case we damage the nose or cause some bleedings. When neprilysin alone was infused then we observed that the number of Aβ depositions increased in the middle and caudal cortex. This was unexpected and it is not easy to explain. While we expected that neprilysin should eliminate the plaques it had the opposite effects. We suggest that intranasal neprilysin rapidly diffused to the brain and rapidly cleared plaques, but this clearance process activated mechanisms in the brains of the transgenic mice to compensate and produce more plaques. These activation mechanisms are not known now. It would be interesting to see if neprilysin could clear the plaques at earlier time points.

### Intranasal Delivery of Neprilysin-Loaded Platelets

As shown, we succeded to load neprilysin into platelets. As platelets are very small anuclear cells, we wanted to take this as an advantage to deliver platelets as a vehicle to allow migration into the brain. In previous studies we delivered platelets via the intravenous route, but failed to find many platelets in the brain, as most of them were trapped in the periphery (lung, spleen) (Kniewallner et al., 2016). Thus in the present study, we wanted to demonstrate that platelets can transmigrate into the brain, via the nasal pathway. In fact, we could show that platelets entered the brain, however, we only could see effects in the frontal cortex and not deeper in the brain. This was expected, as cells may not easily migrate through the brain without being attacked from other immune cells (e.g., microglia). Anyhow, the migration of the platelets was also accompanied by a decrease of plaques in the frontal cortex and subsequent release of neprilysin and we suggest for the first time that neprilysin-loaded platelets are potent vehicles to deliver a drug into the brain.

### Intransal Delivery of Neprilysin Loaded Collagen Hydrogels

In the final step we wanted to take advantage of the collagen hydrogels as a stable delivery tool. Collagen hydrogels were applied directly into the nose on both sides and we expected that they are stable and degrade within a few days and release neprilysin. This application was repeated on 3 consecutive days. In the present study it was not possible to follow up the collagen degradation in the nose and directly proof that bioactive stable neprilysin was released from the collagen. However, our immunostainings clearly show that neprilysin loaded to collagen hydrogels and placed in the nostrils, massively cleared Aβ plaques in the middle and caudal cortex. This effect was not seen in the hippocampal formation. It was surprising that the effect was quite strong and shows for the first time, that collagen-loaded hydrogels are potent intranasal delivery tools. It was also interesting to note that the plaque density in the frontal cortex was not affected. This is not easy to explain, but we suggest a time-dependent process. We hypothesize that neprilysin is slowly released in the nose from collagen hydrogels and in contrast to the pure infusion of neprilysin, it slowly diffuses into the brain. We think that at the observed time point (day 9), neprilysin is active in the caudal cortex, while in the frontal parts again new plaques are produced by the transgenic mouse brain via yet unknown mechanisms. Again an earlier time point could be interesting to test.

### Limits of This Study

This *in vitro* intranasal AD model has definitively several limitations. (1) The most severe limitation is the low neuronal viability of the adult slices, however, as we do not study neurons and only plaques, this issue can be neglected in the present study. (2) As mentioned, the difference between the frontal and caudal parts is not clear. While is may make sense that platelets do not migrate deeper, it is unclear why pure neprilysin enhances plaques in the caudal parts, while collagen-loaded neprilysin massively clears the plaques. It seems to be reasonable that the brain has a very high plasticity and activates (yet unknown) processes to produce more plaques. (3) In our present study we did not follow up degradation of collagen and also not the bioactivity of neprilysin in the nose. We also did not test any negative side effects in the nose (e.g., bleedings), and we also cannot say how the neprilysin enters the brain and which pathways are involved. (4) In our present study, we clearly show that neprilysin has potent stable activity to clear plaques *in vitro*. We also clearly show that intranasal application of neprilysin is very potent to eliminate plaques, and a slow releasing collagen hydrogel has many advantages. Definitely, it would be interesting to see if intranasal delivery of neprilysin clears plaques in human AD patients. (5) If our data are correct and the interpretations point to a compensating mechanism, then the intranasal delivery activates plaque formation after terminating the neprilysin effect. This is of course contra productary and not helpful. Our data first provide evidence that indeed the elimination of plaques can be followed by direct new production of plaques, but it may also indicate that a neprilysin therapy may need a continuous delivery all 2 weeks for several months. This indeed would be an interesting therapeutic approach.

Taken together, in the present study we show that the Aβ-degrading enzyme neprilysin can clear plaques in an organotypic brain slice model of adult transgenic AD mice. We show that neprilysin can be loaded into collagen hydrogels and that intranasal delivery of the neprilysin-loaded collagen hydrogels clears plaques in the caudal cortex *in vivo*. In conclusion, we provide further evidence that neprilysin is a potent drug to clear plaques and that intranasal delivery is a potent method to deliver neprilysin into the brain, however, compensating enhanced plaque production must be considered.

## Data Availability Statement

The raw data supporting the conclusions of this article will be made available by the authors, without undue reservation.

## Ethics Statement

The animal study was reviewed and approved by Austrian Ministry of Science.

## Author Contributions

CH designed and analyzed the data, and wrote the manuscript.

## Conflict of Interest

The author declares that the research was conducted in the absence of any commercial or financial relationships that could be construed as a potential conflict of interest.
